# On the Chiroptical Behavior of Conjugated Multichromophoric Compounds of a New Pseudoaromatic Class: Bicolchicides and Biisocolchicides

**DOI:** 10.1371/journal.pone.0010617

**Published:** 2010-05-12

**Authors:** Tiziana Funaioli, Marino Cavazza, Maurizio Zandomeneghi, Francesco Pietra

**Affiliations:** 1 Dipartimento di Chimica e Chimica Industriale, Università di Pisa, Pisa, Italy; 2 Accademia Lucchese di Scienze, Lettere e Arti, Lucca, Italy; University of Sydney, Australia

## Abstract

**Background:**

It is well known that, stemming from the mutual interplay between chromophores, circular dichroism (CD) is a powerful technique to deal with structural problems for both the small organic molecule and the biopolymer. However, quantitative interpretations of the spectroscopic and structural terms that give rise to the exciton couplet are usually presented for ideal cases, or a few CD bands only are taken into account, overlooking the role of the solvent medium.

**Methodology/Principal Findings:**

Circular dichroism and UV absorption spectra were carried out for colchicide (**3**) and isocolchicide (**6**), as well as their coupling products, 10,10′-bicolchicide (**2**) and 9,9′-biisocolchicide (**5**), in both hydrogen bonding and non hydrogen bonding solvents, as well as MeCN/H_2_O mixtures. A dramatic control by the solvent emerged, as even tiny changes in the composition of solvent mixtures, at *ca* 1 water molar fraction, induced a dramatic modification of their CD bands. A mutarotation phenomenon - long known for isocolchicine (**8**) - was also observed for **5**, and can be attributed to the interconversion between atropisomers (*R*_a_,7*S*),(*R*_a_,7′*S*)-**5a** and (*R*_a_,7*S*),(*S*_a_,7′*S*)-**5b**.

**Conclusions/Significance:**

Our data show that with molecules built on two structurally identical moieties which embody both hydrophilic and hydrophobic groups, even tiny changes in the composition of solvent mixtures cause a dramatic modification of the CD bands. Their analysis arrives at a qualitative rationalization of the observed CD couplets from the coupling of high energy transitions, while attempts at a quantitative interpretation of these phenomena through time-dependent density functional theory allowed to reproduce satisfactorily the CD spectrum in the 300–450 nm region only. Failure with higher energies probably reflects currently inadequate specific theoretical treatments of the solvent medium.

## Introduction

Circular dichroism (CD) is a powerful technique to deal with structural problems for both the small organic molecule and the biopolymer. This stems from largely the mutual interplay between chromophoric units, which affects the molecular properties. Under favorable circumstances, when two or more equivalent chromophores - which absorb light strongly in the same spectral region - are present in a molecular frame at suitable mutual distance and orientation, the CD spectral features (*e.g.* exciton couplets) may offer a clue as to the stereochemistry of the molecule [1], [2].

Models of such systems, built from either classical electrodynamics [3], [4] or quantum mechanics [5], can also allow a quantitative interpretation of the spectroscopic and structural terms that give rise to the exciton couplet [6] [Bibr pone.0010617-Zandomeneghi1]–[8]. However, except in the near-to-ideal cases, success is often limited to a few bands only. In any case, as expected for calculations with electronically excited states, any quantitative interpretation is faced with problems. This is especially true when the conformational behavior of the molecule depends on the nature of the solvent, like for any theoretical treatment of solvent effects.

**Figure 1:**
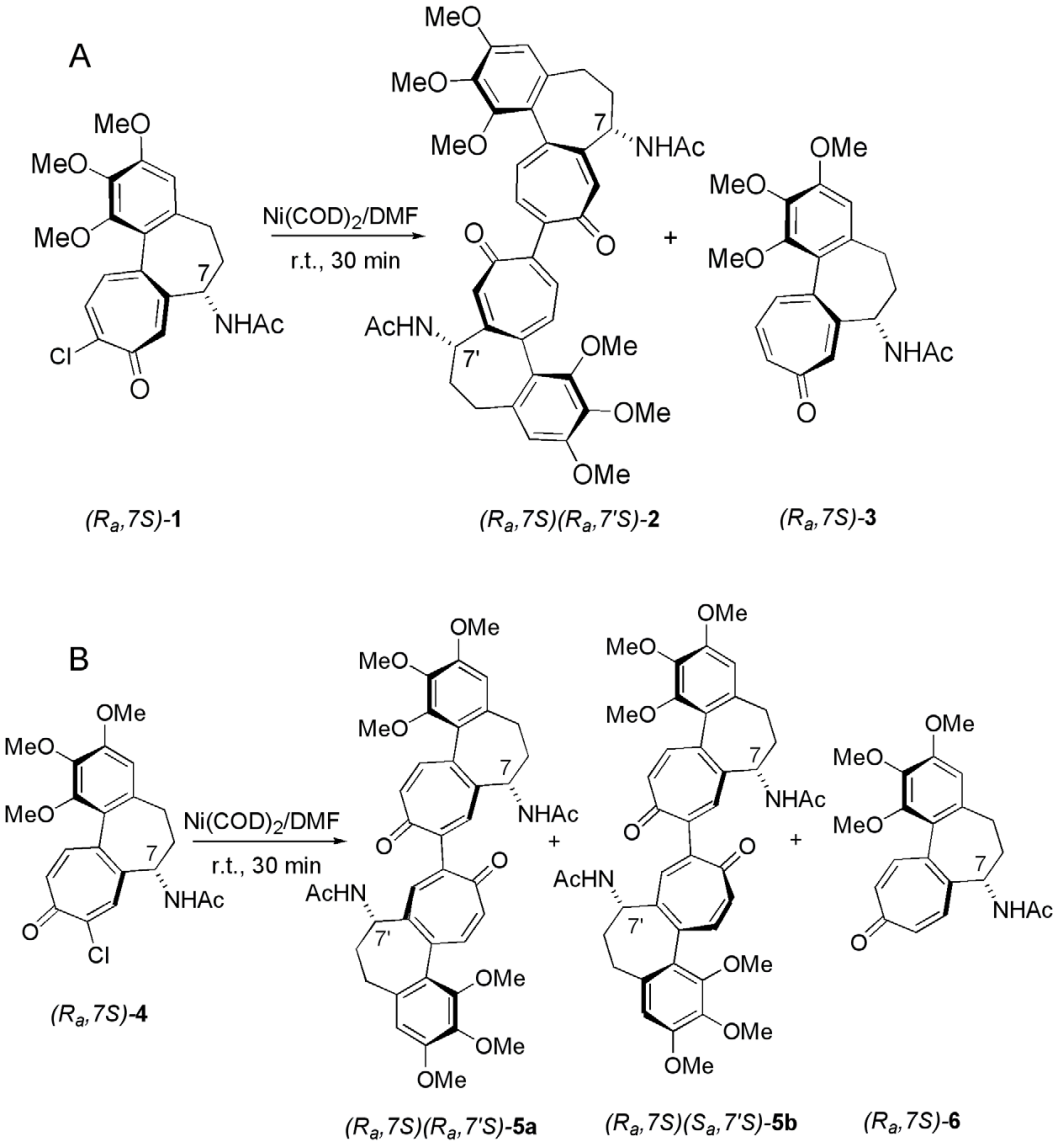
Synthesis of bicolchicide and biisocolchicide. (A) Synthesis of bicolchicide (**2**), (B) synthesis of biisocolchicide (**5**) according to the Semmelhack's method. The stereochemical representations of reagents and products are given.

Here we present a notable example of solvent-dependent circular dichroism involving multichromophoric systems. It concerns 10,10′-bicolchicide (**2**) and 9,9′-biisocolchicide (**5**), where two identical colchicinoid (**1**) or isocolchicinoid (**4**) chromophores of known absolute configuration (*R_a_*,7*S*), are linked through a formally single covalent bond (Figure 1), establishing, as it will become clear in the following, a new pseudoaromatic class. These relatively large molecules (molecular mass 736) embody both hydrophilic and hydrophobic groups, which make them easily soluble in solvents like chloroform, acetonitrile and trifluoroethanol, while being sparingly soluble in water. We illustrate here how the coupling process with **1** or **4**, by increasing the molecular complexity, gives rise to spectroscopic properties that do not show up with the starting molecules.

## Results

### Synthesis of the bicolchicides and biisocolchicides

Compounds **2** and **5** could be obtained by homocoupling of chlorocolchicides by using Semmelhack's recipe, *i.e.*, by inducing the coupling reaction with stoichiometric amounts of Ni(COD)_2_ in DMF at room temperature [9]. This method is further described in Method S1. As shown in Figure 1A for 10-chlorocolchicide (**1**) [10] and Figure 1B for 9-chlorocolchicide (**4**) [10], formation of **2** from the former, and **5** from the latter, was accompanied by hydrogenolysis products, colchicide (**3**) [11] and isocolchicide (**6**) [11], respectively. All efforts to carry out these reactions under catalytic conditions with Ni(COD)_2_ were frustrated. The ^1^H NMR ddd pattern for H7 and H7' supports pseudoaxial assignment for both protons. Since the synthesis started from (*7S*)-**1**, this establishes configuration (*R*_a_,7*S*)(*R*_a_,7'*S*) for compound **2** [12], [13]. For compound **5**, ^1^H NMR spectra at 600 MHz in CDCl_3_ at r.t. revealed that (*R*_a_,7*S*),(*R*_a_,7'*S*)-biisocolchicide (**5a**) and (*R*_a_,7*S*),(*S*_a_,7'*S*)-biisocolchicide (**5b**) equilibrate (83% vs 17%, respectively). These, and all other stereochemical features of these compounds, are dealt with below, step by step, along with spectral evidences.

## Discussion

### UV and CD spectral behavior of bicolchicides and biisocolchicides

**Figure 2:**
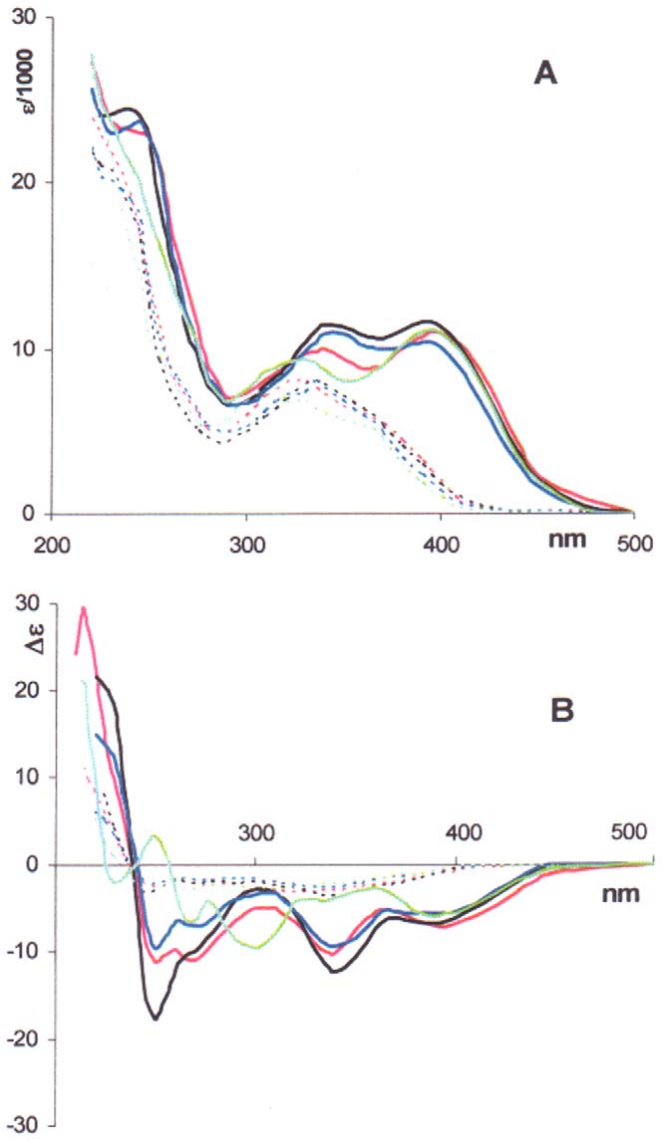
UV and CD spectra for colchicide and bicolchcide. UV (A) and CD (B) spectra for bicolchicide **2** (solid line) and colchicide **3** (dotted line) in various solvents are reported; **^____^**: EtOH; ^____^: TFE; ^____^: H_2_O; ^____^: MeCN.

Figure 2 shows UV and CD spectra in various solvents for the coupling product **2** in comparison with its building block, the hydrogenolysis product **3**. It is seen that the UV and CD bands for **2**, in the low- and medium-energy spectral range (λ>300 nm), undergo a red shift by *ca*. 50 nm with respect to **3**, likely a consequence of conjugation between the two moieties, while gaining in complexity. Extensive conjugation in the dimeric species **2** and **5** is supported by their reduction potential being less cathodic than for the monomeric species **3** and **6**, as determined by cyclic voltammetry. Thus, in dried DMF as solvent, vs SCE, cathodic reduction potentials for compounds **2** and **5** turned out to be -1.28 and -1.27 V, against -1.48 and -1.50 V for compounds **3** and **6**, respectively. (See Method S2.)

Although the CD bands in the low and medium energy spectral region for both **2** and **3** have the same (negative) sign, they differ vastly in both shape and intensity. Notably, **2** is far more sensitive to the nature of the solvent medium than **3**. In the higher energy region (λ<300 nm), the intensity of the CD bands of **2** is even more deeply modulated by the solvent. Thus, with 2,2,2-trifluoroethanol (TFE) and EtOH as solvents, on decreasing the wavelength, the CD of compound **2** attains strongly negative minima, changes its sign at about 240 nm, and acquires positive values at higher energies. In this scenario, the negative and positive CD bands could be considered as branches of an incompletely measured negative exciton couplet. No CD couplet could be observed for **3**.

**Figure 3:**
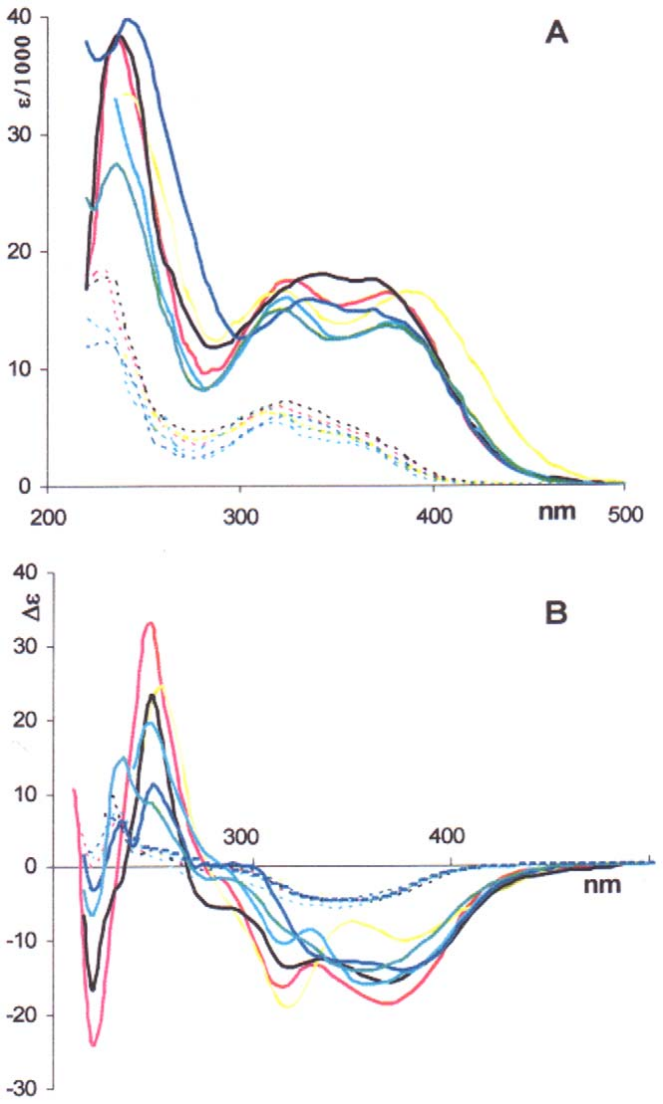
UV and CD spectra for isocolchicide and biisocolchcide. UV (A) and CD (B) spectra for biisocolchicide **5** (solid line) and isocolchicide **6** (dotted line) in various solvents are reported; ^____^: EtOH; ^____^: TFE; **^_^** ^___^: H_2_O; ^____^: MeCN; ^____^: CHCl_3_; ^____^: DMSO.

With both the coupling product **5** (which, as demonstrated below, exists as an equilibrium mixture of two atropisomers **5a** and **5b**, where **5a** highly dominates) and the hydrogenolysis product **6**, a similar trend is observed in the low- and medium-energy spectral range only (Figure 3). Below 300 nm a positive couplet-like band shows up in alcoholic media like EtOH and TFE. In MeCN and water, the couplet-like nature of the CD band is somewhat blurred, while in CHCl_3_ and DMSO recording of CD spectra was limited to 240 nm due to strong absorption by the solvents. Remarkably again, the CD with **5** proved far more solvent dependent than with **6**.

**Figure 4:**
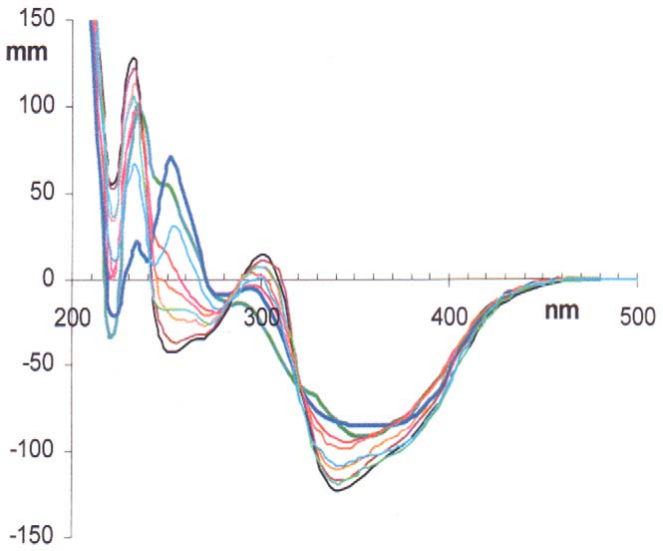
CD spectra for biisocolchicide in MeCN/H_2_O mixtures of various compositions. CD spectra for biisocolchicide **5** in MeCN/H_2_O mixtures of various composition are reported; ^____^: neat H_2_O; ____: neat MeCN (bold lines); elongations are in mm.

Such solvent-induced dramatic changes in the CD bands of the coupling products prompted us to investigate in detail the dependence of the spectral changes from the percentage of MeCN in H_2_O, keeping in mind that the former is a non hydrogen bonding, polar solvent, while the latter is strongly polar, hydrogen bonding [14]. On mixing these wholly miscible solvents, a contraction of volume occurs, in an endothermic process. Out of the plethora of contrasting interpretations or models introduced to explain particular experimental data [14], this process can be simply interpreted as a collapse of some “icebergs” arising from strong hydrogen bonding between water molecules. CD spectra of **5** at constant concentration in MeCN/H_2_O mixtures of nine different compositions - from neat MeCN to neat H_2_O - are shown in Figure 4. These spectral modifications cannot be attributed to any change in the [**5a**]/[**5b**] atropisomeric equilibrium. Actually, we have proven that the [**5a**]/[**5b**] atropisomeric equilibrium is nearly invariant to all compositions of these solvent mixtures, from neat H_2_O to neat MeCN: HPLC analysis (see Materials and Methods section) of the above mixtures shows that the percentage of **5a** in the atropisomeric mixture remains in the range 83–87% throughout. In the light of these observations, the nine dichroic absorption curves in Figure 4 reveal the presence of two isodichroic points at 320 and 280 nm and seven peaks (“extrema”) that could be localized, at least in some recorded CD spectra, at 377, 342, 305, 270, 252, 235, and 222 nm. The presence of isodichroic points, for solutions at constant molarity of the solute, is taken as an indication that the actual spectra derive from a combination of basic spectra having an identical value at the isodichroic points. Quantitative analysis of the data reported in Figure 4, from neat H_2_O to neat MeCN, revealed that the measured CD spectra cannot be expressed as a linear combination of two CD spectra, the one in neat water and the other one in neat MeCN.

**Figure 5:**
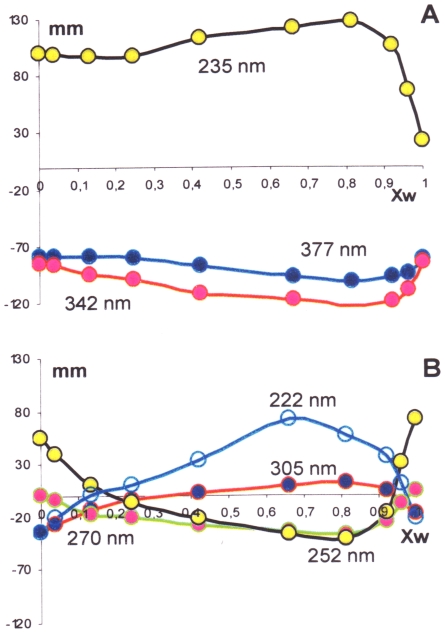
Elongations of the dichroic bands of biisocolchicide with respect to the mole fraction of water. The CD elongations (mm) of 377, 342 and 235 (A) and 305, 270, 252 and 222 nm (B) dichroic bands, derived from the spectra in Figure 4, with respect to the mole fraction of water are reported.

The consequences of changing the percentage of H_2_O in MeCN can be analyzed by considering the intensity of the CD signal at the wavelengths indicated above. This is shown in Figure 5A and 5B, where the dichroic elongations (mm) are given as a function of the mole fraction of H_2_O. The dichroic curves can be grouped into two families. One includes the stronger bands centered at 377, 342 and 235 nm (Figure 5A), where no change in the sign of dichroic bands is observed. In this first family of curves, a very swift change of elongation occurs at vanishingly small concentrations of MeCN, where also thermodynamic ΔH data revealed some peculiarity of the MeCN/H_2_O system [15], whilst further additions of MeCN - up to neat MeCN - have relatively scarce effect on the elongations. Such changes in the elongation probably stem from both the well known disruption ability of OH…O hydrogen bonds and the high affinity for the lipophilic parts of the solute by the solvent MeCN. In this scenario, we envisage the presence of two families of conformers, one related to hydrogen bonded structures (Xw ≫ 0.9 in Figure 5A, at Xw  = 1) and the other one unrelated to hydrogen bonding (Xw <0.9). Associating explicit molecular geometries to these frames is a hard task, however, probably beyond the capability of current continuum solvent models. All groups containing oxygen are first candidates for hydrogen bonding. Included are the methoxy groups (in particular the central one in the 1,2,3-trimethoxybenzene moiety, which is significantly more hydrophilic because it is forced out of the plane of the arene ring [15]), the acetylamino groups, and the carbonyl groups.

The negative bands at 377 and 342 nm must arise from partially conjugated electrons of the two cycloheptatrienone moieties, while the 235 nm band can be interpreted as the positive branch of a partially measured CD couplet. All that must stem from identical aromatic transitions, each one localized in the trimethoxybenzene moiety and coupled to each other according to the known exciton coupling mechanism (see next DeVoe's model section). In both cases, groups such as methoxy (235 nm band) and carbonyl (377 and 342 nm bands) are present in the moiety which is most involved in the transition, hence most strongly influenced by hydrogen bonding.

The conceivable hypothesis of two structural arrangements, one at Xw = 1 and a second one at X_MeCN_  = 1, from which the stronger CD bands centered at 377, 342 and 235 nm originate, does not find a parallel in the analysis of the second family of curves, the weaker dichroic bands at 305, 270, 252 and 222 nm. These, also in the range Xw <0.9, on changing the composition of the solvent undergo marked changes, including the sign of the bands, as it can be appreciated from Figure 5B. More subtly, we are faced by two contrasting observations with these weaker bands. From one side, slight additions of MeCN to aqueous solutions of compound **5** result in steep changes in the elongations, en route to inverting the sign of the bands. From the other side, on adding H_2_O to MeCN solutions of compound **5**, a more gradual change in the elongations is observed in comparison with what occurs when X_w_ is close to 1. As above, the sign of the bands is reversed. This is a more complex trend than was observed with the stronger bands in Figure 5A, where at each wavelength-band two limiting bands were observed on changing from neat MeCN to neat H_2_O. Taking into account the low intensity, the close values of frequency with respect to other intense bands, and the complex mechanisms that exist (*vide infra*) for a transfer of rotational strength from one band to another one, no firm conclusion can be reached at this time about the mechanism underlying the spectral changes with these minor CD bands.

### DeVoe's mechanisms for the origin of circular dichroism from asymmetric locations of chromophores

The onset of exciton couplets suggests that the phenomena described in the previous section are amenable to a rationalization on the basis of DeVoe's classical model [3], [4]. This model is particularly well suited to treat “dimeric” molecules where, like with **2** and **5**, chirality is also contributed by deviations from coplanarity of a couple of identical, or nearly identical, quasi-planar chromophores, *e.g.* the trimethoxy-benzene chromophores. The model can also account for the high sensitivity of CD spectra to relatively minor conformational variations, as it is observed with coupling products **2** and **5**, from what is likely a solvent effect.

Equation (1), which was derived from a coupled oscillator approach [6], expresses the CD due to a series of transitions 1,2,3…i localized in the chromophores of the molecule and coupled through their dipole-dipole interactions. In this frame, transitions related to strongly conjugated electrons, such as those involved in the λ>300 nm spectral range, cannot be considered.

Equation (1), under simplifying physical conditions, can be used to discuss the higher-energy transitions (λ<300 nm) thanks to an explicit expression of the dependency of Δε from both the molecular geometry and the UV spectral features of **1** and **4**:
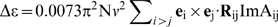
(1)


Here, Im Aij is the imaginary part of a generic element of matrix A, while A is the matrix inverse of matrix B, whose generic element B_ij_ is defined by B_ij_  =  δ _ij_/α_i_ + G _ij_. In this definition, α_i_ is the electric polarizability allied to the i^th^ electronic transition, while G_ij_ (Equation (2)) is the interaction energy between two unit point-dipoles **e**_i_ and **e**_j_. These represent the orientation features of dipole transitions i and j, which are localized in different points of the molecule and are interconnected by the distance **R**_ij_.
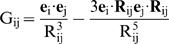
(2)


Because the polarizability is related to the wavelength, matrix inversion has to be made at each value of λ.

A qualitative analysis can be easily carried out under further simplifying conditions, as embodied in Equation (3), which is derived from Equation (1). Equation (3) holds for two identical chromophores with one electrically-allowed transition, in the frame of a treatment first order in the dipole-dipole G_12_ term [6].
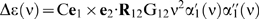
(3)


Here, C is a constant, α_1_′ and α_1_″ are the real and the imaginary parts of polarizability α_1_ pertaining to the transition “1”, and **R**_12_ is the distance between the two dipoles corresponding to the same electronic transition localized in the two molecular moieties.

Two notable features emerge from Equation (3) for the CD generated at the transition “1” spectral range. The first feature is the typical spectral shape of the couplet, which is due to the product of the imaginary part of the polarizability (the α_1_″(ν) term) (which acquires maximum value at the wavelength of maximum absorption, λ_max_) and the real part of the polarizability (the α_1_′(ν) term). The latter, for gaussian or lorentzian spectral shape of α_1_″(ν), undergoes inversion of sign at λ_max_, from positive values at λ > λ_max_, to negative values at λ < λ_max_, following an asymmetric trend with respect to the λ_max_ position. The second feature that emerges from Equation (3) is that the sign and intensity of Δε depend on the molecular geometry, both through the triple mix product of the three vectors that identify the asymmetry of the position/orientation of transition dipoles in the molecule (**e**_l_ , **e**_2_, **R**_12_) and the scalar products of vectors present in the energy term G_12_. Thus, rotation of the two moieties in products **2** and **5** away from a common plane, where Δε  = 0, gives rise to a positive or a negative couplet; the rate of increase of intensity with the deformation parameter turns out to be proportional to both the square of the molar absorption coefficient of the two interacting identical transitions and the dipolar interaction terms. This means that only strong absorption bands can emerge in the CD spectrum with the typical couplet shape. The R_12_ distance between the trimethoxy-benzene moieties is also relevant. It is as high as 14.5 or 13.7 Å for compounds **5a** and **5b**, respectively, in their most stable conformation, as indicated by DFT minimizations (see the computational section). Thus, because of a (1/R_12_)^3^ dependence of G_12_, it is only with particularly favourable orientations of the above three vectors (**e**_l_ , **e**_2_, **R**_12_) that the couplet could emerge.

In the presence of a second transition “2”, not overlapping the one in Equation (3), a term expressed by Equation (4) adds to Δε from the couplet [7].
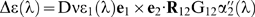
(4)


In Equation (4), D is a constant and “1” represents quantities related to the above described electrically-allowed transition confined to one of the molecular moieties that give rise to the couplet. In turn, “2” represents quantities related to any other transition that does not overlap transition “1” and which is confined to the other molecular moiety. In this case - geometric factor e_1_× e_2_ R_12_ apart - the contribution of transition “2” to the CD in the spectral zone of transition “1” is proportional to the molar coefficient ε_1_(λ) and is positive or negative for absorption band “2” lying at shorter or longer wavelength with respect to band “1”. Terms of this type may induce a shift of the couplet towards positive or negative values, while also causing a deformation of the couplet itself. Joint action of multiple effects of this type can make very difficult to even recognize the presence of a couplet. Probably multiple effects of this type are at work with compounds **2** and **5**, particularly in the case of the bands of Figure 5B, which further complicates the analysis of these bands. When other transitions overlap, even partially, transition “1”, difficulties of interpretation reach the apex.

### Stereochemical assignments of bicolchicide 2 and biisocolchicide 5, and mutarotation with the minor conformer 5b

Other than in the opposite sign of the couplet in the high-energy CD spectral region - as said above - coupling products **2** and **5** differ dramatically in both HPLC chromatographic and ^1^H NMR spectral behavior. While compound **2** showed up, under all circumstances, as a single conformer, the ^1^H NMR spectra of compound **5** in CDCl_3_, at either 300 or 600 MHz, could only be interpreted by disentangling signals for two conformers, **5a** and **5b** (Figure 1), in a 4.8∶1 peak-area ratio. As HPLC analyses of the mixture gave a similar peak-area ratio for **5a** *vs* **5b** (Table 1 and Materials and Methods), the analysis in other solvents was economically carried out from HPLC data alone. The results in Table 1 fail to reveal any trend in the **5a/5b** population ratio with the bulk properties of the medium.

**Table 1:** Experimental distribution of 5a and 5b atropisomers at 300 K.

Method	Medium	ε[a]	5b/5a	ΔG_300_ (Kcal/mol)
^1^H NMR or HPLC	CDCl_3_	4.7	17/83	0.8
HPLC	C_2_H_5_OH	24.3	5/95	1.7
HPLC	CF_3_CH_2_OH	26.5	22/78	0.8
HPLC	CH_3_OH	32.6	6/94	1.7
HPLC	CH_3_CN	36.2	13/87	1.3
HPLC	(CH_3_)_2_SO	49	3/97	2.2
HPLC	H_2_O	78.5	17/83	0.8

[a] Dielectric constant.

**Figure 6:**
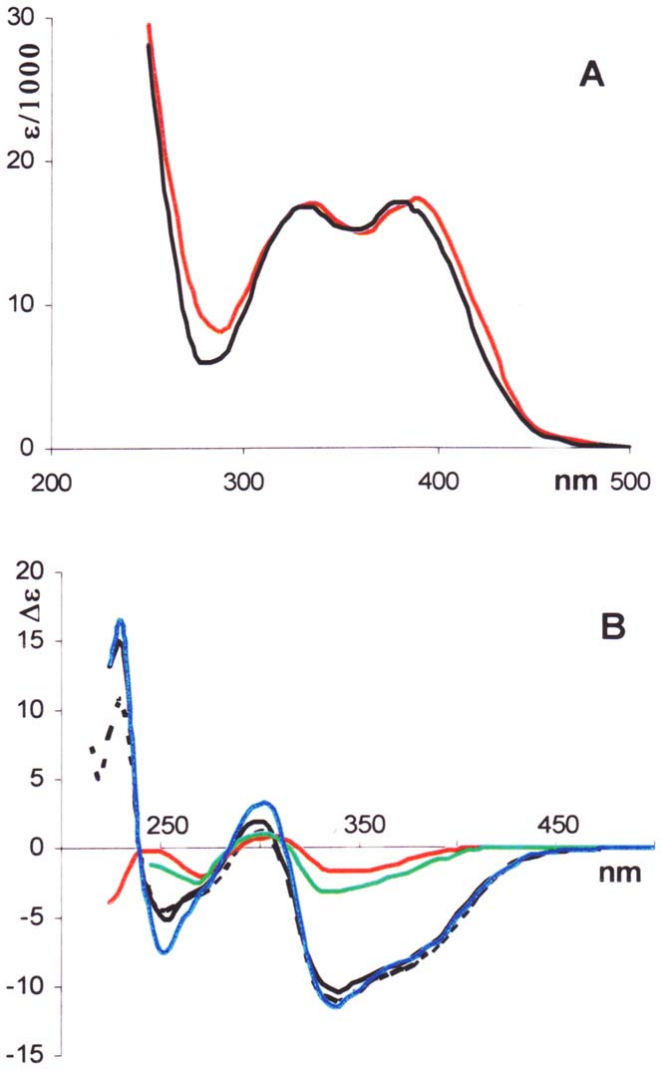
HPLC separation of conformers 5a and 5b. The Figure shows: (A) UV spectra in MeCN/H_2_O 40∶60 for **5a** (^____^) and **5b** (^____^). (B) CD spectra at ca 1°C for **5a** (^____^) and **5b** after 6–30 min (^____^); 95–120 min (^____^); overnight (^____^); finally CD spectrum for the equilibrium mixture **5a**/**5b** at r.t. (-----).

While (*R_a_*,7*S*)(*R*_a_,7'*S*) configurational attribution to **2** and **5a** is based on ^1^H NMR spectra alone (ddd pattern for H7 and H7' for both **2** and **5a**, see Materials and Methods), the stereochemical assignment of the minor conformer **5b** was first carried out on the basis of CD spectra. To do so, mixtures containing compound **5** were subjected to HPLC, peak eluates being directly collected into thermostatted CD cuvettes at *ca* 1°C and rapidly UV and CD analyzed. While the UV spectra proved very similar for all eluates, the dichroism for a minor peak eluted at t_R_  = 8.07 min turned out to increase with time, extrapolating at infinite time (*ca*. 24 h) to the dichroism observed for the major peak (t_R_  = 6.62 min), as shown in Figure 6. Initially, the dichroism in the area centered at λ  = 350 nm was only weakly negative, with elongation much smaller than at infinite time. This is consistent with a partial compensation from the interconnected opposite helices (*R*_a_,7*S*)(*S*_a_,7'*S*).

Configurational assignment (*R*_a_,7*S*)(*S*_a_,7'*S*) to **5b** was confirmed from ^1^H NMR spectra in CDCl_3_, which showed two sets of protons for H7 and H7', differing both as to the chemical shift and the coupling pattern. A ddd pattern supports pseudoaxial assignment to H7 (*i.e.* the C7 acetamido group takes a pseudoequatorial position) for the *R*_a_ part, while a dd pattern supports pseudoequatorial assignment to H7' (*i.e.* the C7 acetamido group takes a pseudoaxial position) for the *S*_a_ part [12], [13].

**Figure 7:**
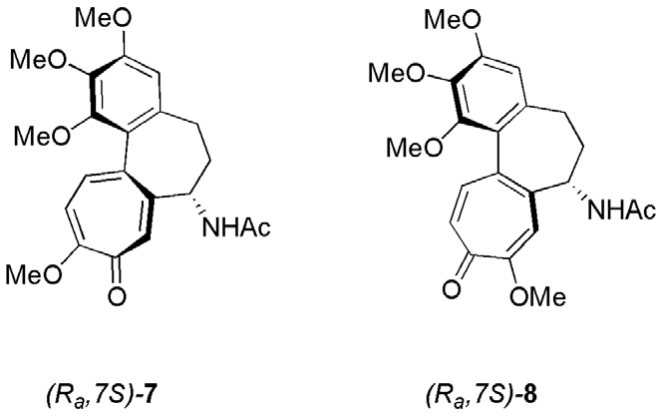
Structure of colchicine and isocolchicine. These structures represent the(*R*_a_,7*S*) configuration of both colchicine (**7**) and isocolchicine (**8**).

The time dependency observed for the dichroism of compound **5** must stem from mutarotation, with the minor conformer (*R*_a_,7*S*)(*S*_a_,7'*S*)-**5b** undergoing helical inversion to give rise to (*R*_a_,7*S*)(*R*_a_,7'*S*)-**5a**. The equilibrium constant K**_(_**_***S*a)-5/(*R*a)-5**_ between the two atropisomers is sensitive to the solvent nature (Table 1), and, like for the CD spectra of compounds **2** and **5**, the changes can hardly be related to the bulk properties of the medium. Moreover, the trend observed for the separate moieties (let them be represented by the known colchicine (**7**) and isocolchicine (**8**), Figure 7), is also not followed.

Rapoport and Lavigne first rationalized the mutarotation of **8** in chloroform as due to atropisomeric inversion [16]. They also attributed lack of mutarotation of isocolchicine in ethanol to increased steric bulk of the ethanol-solvated pseudoaxial acetylamino group [16]. Although lack of mutarotation for isocolchicine in ethanol is also consistent with favored solvation of the more exposed pseudoequatorial acetylamino group stabilizing this conformation [17], whichever rationalization applies to this solvent effect, the behavior of the “dimeric” structures, resulting from coupling of either two colchicides, or two isocolchicides, is formally in line with their “monomeric” components. To this concern, it should be noticed that dominance of the (*R*_a_,7*S*)(*R*_a_,7'*S*)-**5a** atropisomer by 4.8∶1 over the (*R*_a_,7*S*)(*S*_a_,7'*S*)-**5b** atropisomer in CHCl_3_ at r.t. is less than observed (10∶1) for the (*R*_a_,7*S*) *vs* the (*S*_a_,7*S*) atropisomer of isocolchicine **8** [17]. That is, “dimerization” smoothes out any difference between the equilibrating atropisomers. That said, it should be noticed that the CD spectra of the ensemble (Figure 3B) depend more on solvent effects on conformer **5a** than on the position of the equilibrium of this conformer with **5b**, in spite of profound differences that exist in the dichroism of the two conformers, **5a** and **5b** (Figure 6B).

### Computational treatment of bicolchicides and biisocolchicides

**Figure 8:**
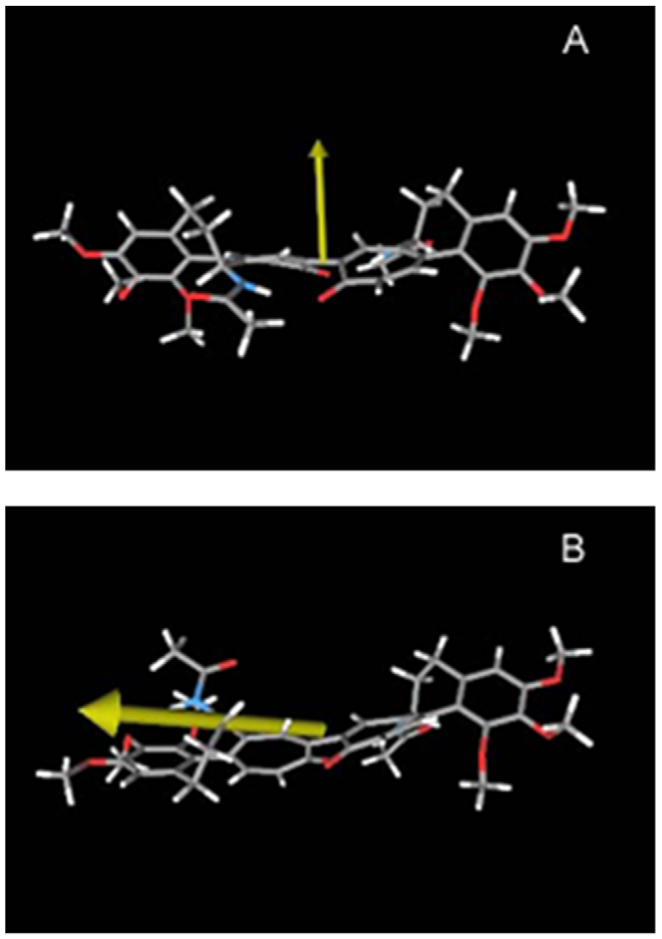
Geometry optimized structures of biisocolchicide atropisomers. The geometry optimized structures (level DFT/M05-2X/6-31G*, *in vacuum*) of biisocolchicide atropisomers (*R*_a_,7*S*)(*R*_a_,7'*S*)-**5a** (A) and (*R*_a_,7*S*)(*S*_a_,7'*S*)-**5b** (B) are shown. The yellow arrow indicates the direction of the dipole moment.

Structure **5** was minimized *in vacuum* by both global space search with molecular mechanics [18] and simulated annealing molecular dynamics [19]. Three types of conformers, (*R*_a_,7*S*)(*R*_a_,7'*S*), (*R_a_,7S)(S_a_,7'S*), and (*S_a_,7S*)(*S_a_,7'S*) emerged. While the latter one is of no interest for its high potential energy, the lowest-energy conformers of the other two types were further minimized by DFT calculations with the M05-2X functional [20] at 6–31G* basis set level (see Calculations S1). The choice of this recent density functional was dictated by its correct treatment - even at this modest basis set level - of the component moieties, colchicine (**7**) and isocolchicine (**8**), where the B3LYP density functional resulted in a much too high puckering of the cycloheptatrienone ring [17]. Success of M05-2X could be attributed to a better treatment of medium-range correlations than by B3LYP [20]. As shown in Figure 8, with both (*R*_a_,7*S*)(*R*_a_,7'*S*)-**5a** and (*R*_a_,7*S*)(*S*_a_,7'*S*)-**5b** the cycloheptatrienone ring was simulated correctly by using the M05-2X functional, with only slight puckering in accordance with X-ray diffraction data for colchicinoids [21], [22] or nematic-phase NMR for tropone [23]. According to these DFT calculations, atropisomer **5a** is more stable than **5b** by 2.5 kcal mol^−1^. Single point MP2 energy calculations at the same set of basis level, which better account for electron correlations, **5a** turned out to be more stable than **5b** by a smaller margin, 1.8 kcal mol^−1^. Still, this is a larger margin than observed experimentally, with the exception of experiments in DMSO as solvent, where calculated values match experimental values (Table 1). However, in view of the lack of any trend of the experimental energy with the solvent properties (Table 1), the agreement observed for DMSO as solvent should be considered as fortuitous. In view of these facts, the coupling product **5** represents a more challenging system than either **8** or **7**, where the energy predictions by DFT or MP2 *in vacuum* are in good agreement with the experimental data in CHCl_3_ as solvent [16]. Likely, this reflects the higher complexity of **5**.

**Figure 9:**
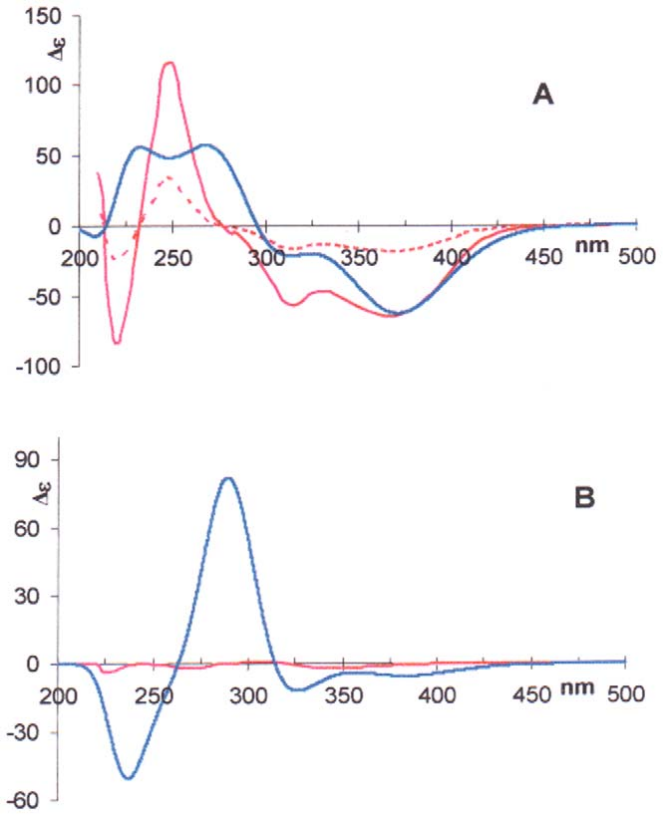
Calculated CD spectra of 5a and 5b. (A) CD spectra of **5a**. -----: experimental spectrum in EtOH; ^____^: the same multiplied by three; ^____^: calculated spectrum in EtOH, σ = 0.32. (B) CD specta of **5b**. ^____^: experimental spectrum in MeCN/H_2_O 40∶60; ^____^: calculated spectrum in EtOH, σ = 0.32.

Quantum mechanical simulation of the UV and CD spectra of atropisomers **5b** and **5a** was also carried out in the frame of the time-dependent density functional theory (TDDFT), by using BHLYP, a hybrid functional with a large amount of Fock-exchange [24], with TZVP as basis set. Because the RI-DFT mode was used, the auxiliary basis set TZV/J had to be added. It is seen from Figure 9A for atropisomer **5a** that the low-energy bands only (λ >300) were decently reproduced. It can also be noticed that a plotting common artifice, broadening the CD bands with σ  = 0.32, seems to afford a better fitting with respect to narrower bands, *e.g.* σ  = 0.16 [25]. Actually, the substance of the simulation does not change: the bands at higher energy (λ<300) could not be reproduced. With atropisomer **5b**, the two moieties interconnected at the cycloheptatrienone rings have opposite helicity (Figure 1B). This leads to only weak (negative) CD (Figure 9B). Our simulations are in accordance for the low-energy zone (λ>300), while, like for **5a**, the high-energy zone (λ<300) could not be reproduced.

As far as solvent effects are concerned, it must be said that DFT calculations with continuum solvation models, such as devised by Klamt [26], Caricato [27], Cramer and Truhlar[28], and Florián and Waershel [29], do not attain a precision sufficient to deal with the tiny conformational energy differences with atropisomers **5a** and **5b** and therefore were not even considered for present tasks. MD procedures in explicit solvent could be carried out in a periodic system, also with pseudopotential plane-wave density functional theory, such as implemented in the NWChem suite [30], or in linear-scaling implementations [31], where parameterization is carried out on-the-fly. A limitation to this theory can be seen in the requirement of trajectories long enough to reach convergence, which would be computationally too costly for molecules of the size of bicolchicides. More fundamentally, the currently implemented Becke-Lee-Yang-Parr (BLYP) density functional [31] is inadequate to treat the cycloheptatrienone nucleus, where even the more advanced hybrid functional B3LYP failed [17]. On the other hand, any treatment of biisocolchicide conformers by classical MD would require to build an *ad hoc* high-level force field, without any guaranty of success given the tiny energy differences into play.

What remains open to question, are QM-MM calculations, where the solute is treated quantum mechanically, while the solvent is treated classically [32]. A foreseeable problem here is that MD procedures are characterized by extensive sampling in view of long-enough trajectories, in the order of many ns so that, to alleviate the burden of the big matrices, QM-MM procedures of the type required in this work are currently carried out with a semiempirical level for the QM part [32], thus inheriting all limitations of semiempirical treatments. Nonetheless, we tried this route. Thus, PM3 was attempted in explicit solvents with Amber 10 suite and GAFF force field [33], resulting in an unrealistic strong puckering of the cycloheptatrienone rings and unreliable energy output. On the other hand, the SCC-DFTB level [34], attempted *in vacuum*, led to similarly disappointing results.

### Conclusions

A central observation in this work is that DeVoe's model for circular dichroism [3], [4] is very important. In fact this model allows us to perform a successful qualitative analysis of the dichroic behaviour of both **2** and **5**. For any quantitative application of DeVoe's model [3], [4] prospects are far less alluring, however. In order that the model retains validity in quantitative applications, the electronic transitions on the component moieties of our coupling products should be independent from one another, while with compounds **2** an **5** it is expected that DeVoe's model is invalidated by substantial conjugation between the component moieties. For the bands at higher energy, in particular those involved in exciton couplets, the number, wavelength features, and polarization direction of the transitions need to be accurately known in order that the CD spectrum can be predicted in the frame of DeVoe's model. This would require a reliable deconvolution of the absorption spectrum for the moieties that constitute the coupling products **2** and **5** into the component bands, as well as the measurement of polarization directions of related transitions. Moreover, the geometrical relationships between the moieties in the global molecule should be accurately known in view of the sensitivity of the CD to even small changes in the relative orientation of transition dipoles localized at each trimethoxy-benzene moiety. These changes may turn out to be cumulative effects of small distortions along the molecular backbone, of neat significance for equations (1–4).

All that draws attention to four key points: (1) the trimethoxy-benzene chromophores are separated by a large distance, ca. 14 Å, (2) they are interconnected by flexible moieties that are exposed to the solvent medium, (3) the CD is highly sensitive both to the relative orientation of these chromophores and (4) the many existing transitions which can modify the couplet profile. Thus, CD couplets can only emerge clearly when favorable orientations of the transition dipoles are met, which, unsurprisingly, can only happen for certain solvents. All that should be considered in the above illustrated framework, *i.e*., that for multichromophoric molecules the interplay of various factors in the interaction with circularly polarized light may face such a delicate balance of factors that the presence of the couplet may even escape attention.

Our limited success in the quantum mechanical simulation of the CD of atropisomers **5a** and **5b** reflects general obstacles encountered in such computations, whose importance in any real case is difficult to assess. These include basis set non-completeness (which, admittedly, could be ameliorated, albeit at much increasing computational cost), difficulty in thoroughly accounting for electron correlation, zero-point vibrations, and any conceivable tunneling between multiple energy minima for the different conformations that are very close in energy [35]. Unavailability of adequate models to treat specific interactions of the solvent with organic molecules is another major obstacle, which makes the reliability of computational treatments more and more questionable as the molecule increases in complexity. In our case, on going from colchicides **3** and **6** to coupling products **2** and **5**, the borderline between what can be treated and what cannot was surpassed. Our analysis of the CD spectra of “monomers” and “dimmers” in various solvents revealed that specific account is needed for each solvent used, a task that we found hard to accomplish. In practice, this borderline in tractability is set by both the characteristics of the CD spectra in solvents of varying properties and by the computational resources. At any event, our study shows that understanding optically active molecules of the complexity of our coupling products needs, from the experimental side, acquiring CD spectra in different solvents, and, from the computational side, establishing and using methodologies able to account for *specifically* each solvent used.

Our work shows that obstacles faced by the theoretical interpretation of CD spectra get up dramatically. Besides widely documented general difficulties in predicting CD spectra [35], the main asperities at the structural level are identified here in the presence of both hydrophobic and hydrophilic groups in the same molecule, where chirality arises more from the helicity than the asymmetric carbon [12]. This is exacerbated, with respect to the simpler colchicinoids and isocolchicinoids, by the presence of a further node in the helix, at the central bond that connects the two moieties, the importance of which is difficult to predict in condensed phase. All that gives rise to spectral complexities that pose challenges to computational theories and justify establishing for our coupling products a new class of pseudoaromatic compounds.

## Materials and Methods

### Materials

Spectroscopic-grade EtOH, MeOH, MeCN, and DMSO (C. Erba), as well as Ni(COD)_2_ (Aldrich), were used as such. DMF (C. Erba) was distilled from CaO and stored over 30 µm molecular sieves under Ar. 2,2,2-Trifluoroethanol (Aldrich) was distilled before use. 10-Chlorocolchicide (**1**) and 9-chloroisocolchicide (**4**) were prepared according to literature [10].

### Techniques

UV-VIS: Perkin-Elmer Hitachi 200; CD: Jasco J-40AS; IR: Perkin-Elmer Spectrum One FT-IR. ^1^H NMR: Varian Gemini BB 200, Unity 300, and Inova 600 MHz spectrometers, with TMS as internal reference, with *J* values in Hz. Mass spectra: Applied Biosystems Sciex API 4000, MDS Sciex, Concord, Ontario, Canada, triple quadrupole mass spectrometer equipped with a Turbo-V Ionspray source coupled to a Perkin Elmer Series 200 Micro Pump by Flow Injection. Analysis: 200 µl/min under the following experimental conditions: CUR, 10; GS1, 25; GS2, 25; IS Voltage, 5 kv, Turbo T, 300°C; DP, 20 V. HPLC: Jasco Uvidec-100-V with SPD-10A Shimadzu UV-VIS detector; column1: Xper-Chrom C18 5 µm 250×4.6 mm; column 2: Technicol Kromasil C18 250×10 mm, eluent MeCN/water 40∶60.

### Synthesis of 10,10′-bicolchicide (2)

Starting from 10-chlorocolchicide (**1**) (Figure 1A), 10,10′-bicolchicide (**2**) (57.8% yield) was obtained as a yellow-orange gummy solid alongside colchicide (**3**) [11] (26.2% yield). Spectral data of **2**: ^1^H NMR (300 MHz, CDCl_3_, 25°C, TMS): δ  = 7.45 (d, 2H, H12, H12′, *J*  = 9.6) , 7.37 (s, 2H, H8, H8′), 7.23 (d, 2H, H11, H11′, *J*  = 9.6), 6.96 (d, 2H, NH, *J*(NH,7)  = 6), 6.52 (s, 2H, H4, H4′), 4.69 (ddd, 2H, H7 and H7′, *J*(7,NH)  = 6, *J*  = 6.6, *J*  = 12), 3.93, 3.89 and 3.70 (three s, 18 H, OCH_3_), 2.2–2.5 (m, 8H, H5, H6, H5′,H6′), 2.0 (s, 6H, COCH_3_). See Figure S1. ^13^C NMR (50.3 MHz, CDCl_3_, 25°C, TMS): δ  =  23.1, 29.9, 36.5, 51.8, 56.2, 61.3, 61.7, 107.6, 125.5, 131.5, 134.2, 135.1, 135.6, 143.2, 150.1, 151.5, 153.8, 169.5, 184.6. See Figure S2. IR(neat): ν  = 1660, 1615, 1556, 1511, 1456, 1403, 1347, 1320, 1260. ESI-MS: m/z  = 737.3 [M + H]^+^, 759.4 [M + Na]^+^. HRMS: calcd for C_42_H_45_N_2_O_10_ 737.3069; found 737.3065.

### Synthesis of 9,9′-biisocolchicide (5)

Starting from 9-chloroisocolchicide (**4**) (Figure 1B), 9,9′-biisocolchicide (**5**) (67.1% yield) was obtained as a yellow-orange gummy solid alongside isocolchicide (**6**) [11] (10.4% yield). Spectral data of **5a**: ^1^H NMR (600 MHz, CDCl_3_, 25°C, TMS): δ  = 8.54 (d, 2H, NH, *J*(NH,7)  = 7.8), 7.52 (s, 2H, H8, H8′), 7.35 (d, 2H, H12, H12′, *J*(12,11)  = 12.6), 7.05 (d, 2H, H11, H11′, *J*(11,12)  = 12.6), 6.56 (s, 2H, H4, H4′), 4.44 (ddd, 2H, H7, H7′, *J*(7,NH) * = *7.8, *J_7,_*_pro(R)6_  = 6.6, *J_7_*_,pro(S)6_  = 12.6), 3.89, 3.85 and 3.72 (three s, 18 H, OCH_3_), 2.47 (dd, 2H, pro(S)H5, pro(S)H5′, *J*_pro(S)5,pro(R)5_  = 12.5, *J*_pro(S)5,pro(R)6_  = 6.5), 2.38 (ddd, 2H, pro(R)H5, pro(R)H5′, *J*_pro(R)5,pro(S)5_  = 12.5, *J*_pro(R)5,pro(R)6_ = 13, *J*_pro(R)5,pro(S)6_  = 6.5), 2.27 (dddd, 2H, pro(R)H6, pro(R)H6′, *J*_pro(R)6,pro(S)6_  = 12.5, *J*_pro(R)6,pro(S)5_  = 6.5, *J*_pro(R)6,pro(R)5_  = 13, *J*_pro(R)6,7_  = 6.6), 2.18 (ddd, 2H, pro(S)H6, pro(S)H6′, *J*_pro(S)6,pro(R)6_  = 12.5, *J*_pro(S)6,pro(R)5_  = 6.5, *J*_pro(S)6,7_  = 12.6), 1.94 (s, 6H, COCH_3_). Spectral data of **5b**: ^1^H NMR (600 MHz, CDCl_3_, 25°C, TMS): δ  = 7.62 (s, 1H, H8′), 7.47 (s, 1H, H8), 7.40 (d, 1H, H12, *J*(12,11) = 12.6), 7.30 (d, 1H, H12′, *J*  = 12.6), 7.06 (d, 1H, H11 or H11′, *J*(11,12)  = 12.6), H11 or H11′ overshadowed by H11 protons of **5a**, 6.20 (s, 1H, H4′), 6.57 (s, 1H, H4), 5.17 (d, 1H, NH′, *J*_NH,7′_  = 7.8), 4.92 (dd, 1H, H7′, *J*_7,NH_ *= *7.8, *J_7,_*_pro(R)6_  = 7.8), 4.20 (ddd, 1H, H7, *J*_7,NH_ *= *7.8, *J _7,_*_pro(R)6_  = 6.6, *J_7_*_,pro(S)6_  = 12.6), 3.95, 3.93, 3.91, 3.90, 3.76, 3.70 (six s, 18 H, OCH_3_ and OCH_3_'), 2.74 (dddd, 1H, pro(R)H6′, *J*_pro(R)6′,pro(S)6′_  = 14.5, *J*_pro(R)6′,pro(S)5′_  = 12, *J*_pro(R)6′,pro(R)5′_  = 6, *J*_pro(R)6′,7′_  = 7.8), 2.59 (ddd, 1H, pro(S)H5′, *J* not given), other H5, H6, H5′ and H6′ overshadowed by corresponding protons of **5a**, 2.17 (s, 3H, COCH_3_), 1.62 (s, 3H, COCH_3_'). See Figure S3. For **5b**, protons labeled by a quotation mark pertain to the molecular moiety with axial configuration *S*_a_. These assignments are supported by 2D proton-COSY experiments in CDCl_3_. ^13^C NMR (50.3 MHz, CDCl_3_, 25°C, TMS): δ  = 23.3, 29.9, 38.8, 53.0, 56.4, 61.4, 108.1, 128.7, 133.8, 135.4, 136.4, 138.1, 140.0, 144.5, 150.8, 154.3, 170.4, where the two atropisomers cannot be distinguished because of nearly matching signals. See Figure S4. IR(neat): ν  = 1652, 1610, 1587, 1556, 1489, 1454, 1403, 1347, 1321. ESI-MS: m/z  = 737.3 [M + H]^+^, 759.4 [M + Na]^+^. HRMS: calcd for C_42_H_45_N_2_O_10_ 737.3069; found 737.3069.

### Equilibration of atropisomers and CD spectra

HPLC (column 1, flow rate 1 mL/min, λ 330 nm) of a CHCl_3_ solution of compound **5** showed two peaks with retention times 5.65 min (**5a**) and 6.88 min (**5b**) in a relative peak-area ratio 4.9∶1. The two atropisomers gave nearly superimposable UV spectra (SPD-10A Shimadzu UV-VIS detector) (Figure 6). The same composition was obtained by collecting eluates at 6.82 and 8.07 min from preparative HPLC (column 2, flow rate 4 mL/min, λ 330 nm), followed by CHCl_3_ extraction and drying. Following rapid evaporation to dryness under vacuum, and addition of an equal volume of EtOH, HPLC analysis showed that the **5b/5a** abundance ratio had risen from initial 17∶83 to 5∶95 in 45 min at 22°C. On evaporation of EtOH to dryness, and addition of an equal volume of CHCl_3_, the abundance ratio **5b/5a** ratio was restored to 17∶83 in 150 min at 22°C. A similar behavior was observed in other solvents, as shown in Table 1. Preparative HPLC collection of eluates at the 6.82 and 8.07 min peaks into precooled cuvettes allowed us to record the CD spectra at *ca* 1°C for atropisomers **5a** and **5b** in MeCN/H_2_O 40∶60 (Figure 7). The CD spectrum of **5b** was observed to change with time into a spectrum for the equilibrium **5a/5b** mixture, the equilibrium being reached overnight. The initial rate of **5b → 5a** transformation could be estimated as ΔCD elongation at 350 nm/Δt  = 0.044 cm/min at 1°C.

### Theoretical methods used

Global conformational space search was carried out with program GMMX, based on Steliou's BAKMDL algorithm, which involves a systematic variation of bond lengths, angles, and formal breaking-closure of rings. Force field MMX [18] was used. Repetitive global space search, forth and back, and from different intermediate positions, converged to the same minima, which should therefore be close to the global minimum. Simulated annealing was carried out with software AMBER with GAFF force field [33], driven by a Python script [19]. DFT calculations were carried out with the suite NWChem [30]. TDDFT calculations were carried out with software ORCA [36]. Analysis and plotting of the results was carried out with software SpecDis v1.45 [25].

## Supporting Information

Method S1 Homocoupling of halocolchicides.
(0.03 MB DOC)

Method S2 Cathodic reduction potentials of colchicine, colchicides and bicolchicides.
(0.02 MB DOC)

Calculations S1 Quantum mechanical calculations; molecular dynamics.
(0.09 MB DOC)

Figure S1 ^1^H NMR spectrum (300 MHz, CDCl_3_) for compound 2.
(0.11 MB TIF)

Figure S2 ^13^C NMR spectrum (75 MHz, CDCl_3_) for compound 2.
(0.15 MB TIF)

Figure S3 ^1^H NMR spectrum (600 MHz, CDCl_3_) for 5a (83%) and 5b (17%).
(0.14 MB TIF)

Figure S4 ^13^C NMR spectrum (75 MHz, CDCl_3_) for 5a (83%) and 5b (17%).
(0.15 MB TIF)
