# Correction: On the Chiroptical Behavior of Conjugated Multichromophoric Compounds of a New Pseudoaromatic Class: Bicolchicides and Biisocolchicides

**DOI:** 10.1371/annotation/c8a83120-e963-40f4-8591-90bd7f0c6b37

**Published:** 2010-07-09

**Authors:** Tiziana Funaioli, Marino Cavazza, Maurizio Zandomeneghi, Francesco Pietra

There was an error in the figure legends for this manuscript. Please refer to the figure legends below.

Figure 1. Synthesis of bicolchicide and biisocolchicide. (A) Synthesis of bicolchicide (2), (B) synthesis of biisocolchicide (5) according to the Semmelhack's method. The stereochemical representation of reagents and products is given.

Figure 2. UV and CD spectra for colchicide and bicolchcide. UV (A) and CD (B) spectra for bicolchicide 2 (solid line) and colchicide 3 (dotted line) in various solvents are reported; red line: EtOH; black line: TFE; blue line: H2O; green line: MeCN.

Figure 3. UV and CD spectra for isocolchicide and biisocolchcide. UV (A) and CD (B) spectra for biisocolchicide 5 (solid line) and isocolchicide 6 (dotted line) in various solvents are reported; red line: EtOH; black line: TFE; blue line: H2O; green line: MeCN; yellow line_: CHCl3; sky-blue line: DMSO.

Figure 4. CD spectra for biisocolchicide in MeCN/H2O mixtures of various compositions. CD spectra for biisocolchicide 5 in MeCN/H2O mixtures of various composition are reported; blue line: neat H2O; green line: neat MeCN (bold lines); elongations are in mm.

Figure 5. Elongations of the dichroic bands of biisocolchicide with respect to the mole fraction of water. The CD elongations (mm) of 377, 342 and 235 (A) and 305, 270, 252 and 222 (B) dichroic bands, derived from the spectra in Figure 4, with respect to the mole fraction of water.are reported.

Figure 6. HPLC separation of conformers 5a and 5b. The Figure shows: (A) UV spectra in MeCN/H2O 40:60 for 5a (black line) and 5b (red line). (B) CD spectra at ca 1&deg;C for 5a (blue line) and 5b after 6-30 min (red line); 95-120 min (green line); overnight (black line); finally CD spectrum for the equilibrium mixture 5a/5b at r.t. (dotted line).

Figure 7. Structure of colchicine and isocolchicine. These structures represent the(Ra,7S) configuration of both colchicine (7) and isocolchicine (8).

Figure 8. Geometry optimized structures of biisocolchicide atropisomers. The geometry optimized structures (level DFT/M05-2X/6-31G*, in vacuum) of biisocolchicide atropisomers (Ra,7S)(Ra,7'S) 5a (A) and (Ra,7S)(Sa,7'S) 5b (B) are shown. The yellow arrow indicates the direction of the dipole moment.

Figure 9. Calculated CD spectra of 5a and 5b. (A) CD spectra of 5a. Red dotted line: experimental spectrum in EtOH; red line: the same multiplied by three; blue line: calculated spectrum in EtOH, sigma=0.32. (B) CD specta of 5b. Red line: experimental spectrum in MeCN/H2O 40:60; blue line: calculated spectrum in EtOH, sigma=0.32.

