# Supplementary material for: On the Chiroptical Behavior of Conjugated Multichromophoric Compounds of a New Pseudoaromatic Class: Bicolchicides and Biisocolchicides
Source: PLoS One. 2010 May 12;5(5):e10617. doi: 10.1371/journal.pone.0010617 (PMC2868894; doi:10.1371/journal.pone.0010617)
Supplement: Method S1 — Homocoupling of halocolchicides. (0.03 MB DOC) [file pone.0010617.s001.doc]

Homocoupling of halocolchicides

**General Remarks.** Toluene (C. Erba) was distilled from Na under an atmosphere of Ar. DMF (C.Erba) was distilled from CaO and stored over 30 μm molecular sieves under an atmosphere of Ar. Ni(COD)_2_ (Aldrich) was used as such. 10‑Chlorocolchicide (**1**) and 9‑chloroisocolchicide (**4**) were prepared according to literature [10]. Preparative TLC: 20x20 cm silica gel Analtech plates.

**Homocoupling of halocolchicides promoted by bis(1,5-cyclooctadiene)nickel in DMF (Semmelhack methodology** [9])

***N-*(7′-Acetylamino-1,2,3,1′,2′,3′-hexamethoxy-10,10′-dioxo-5,6,7,10,5′,6′,10′-octahydro-[9,9′]bi[benzo[*a*]heptalenyl]-7-yl)-acetamide (diisocolchicide 5)** (**as a mixture of atropisomers**)

9‑Chloroisocolchicide (**4**) (0.550 g, 1.36 mmol) was added to a stirred solution of Ni(COD)_2_ (0.268 g, 0.97 mmol) in 33.5 mL of DMF under an atmosphere of Ar. After 30 min at r.t., the dark green solution was evaporated under vacuum to give a gummy residue that was subjected to silica‑gel TLC, eluting with CHCl_3_/MeOH 9:1. The yellow band at R_f_ = 0.53 was repeatedly extracted with CHCl_3_ to give compound **5** as an orange yellow solid (0.336 g, 0.456 mmol, 67.1% yield). Isocolchicide (**6**) (0.052g, 0.14 mmol, 10.4% yield) was recovered from a colorless band at R_f_ = 0.64. Analytical data for compound **6** agreed with literature [11].

^1^H NMR (DMSO, 200 MHz) revealed substantially pure **5a** at equilibrium ( 97%, see Table 1, text): 8.38 (d, 2H, NH, *J* = 7.2 Hz), 7.32 (s, 2H, H8, H8'), 7.11 (d, 2H, H12, H12' *J* = 12.7 Hz), 6.79 (d, 2H, H11, H11', *J* = 12.7 Hz), 6.74 (s, 2H, H4, H4'), 4.27 (td, 2H, H7, H7' *J =* 7, *J* = 6, *J* = 10 Hz), 3.75, 3.70 and 3.57 (three s, 18 H, OCH_3_), 1.8-2.2 (m, 8H, H5, H6, H5', H6'), 1.72 (s, 6H, COCH_3_). For the separation and the spectral data of both **5a** and **5b** see the Experimental part.

**(*R*_a_*,*7*S*)(*R*_a_*,*7′*S*)-*N-*(7′-Acetylamino-1,2,3,1′,2′,3′-hexamethoxy-9,9′-dioxo-5,6,7,9,5′,6′,9′-octahydro-[10,10′]bi[benzo[*a*]heptalenyl]-7-yl)-acetamide (dicolchicide 2)**

Prepared as described above for compound **5**, starting from 10‑chlorocolchicide (**1**) (0.167 g, 0.414 mmol), Ni(COD)_2_ (0.081 g, 0.294 mmol) in 11 mL of DMF. Silica‑gel TLC, eluting with CHCl_3_/MeOH 9:1, gave compound **2** (0.088 g, 0.12 mmol, 57.8 % yield) at R_f_ = 0.37 and colchicide (**3**) (0.040 g, 0.108 mmol, 26.2 % yield) at R_f_ = 0.54. Analytical data for compound **3** agreed with literature [11]. HPLC analysis gave only one peak for compound **2**. For the spectral data of **2** see the Experimental part.
