# Supplementary material for: On the Chiroptical Behavior of Conjugated Multichromophoric Compounds of a New Pseudoaromatic Class: Bicolchicides and Biisocolchicides
Source: PLoS One. 2010 May 12;5(5):e10617. doi: 10.1371/journal.pone.0010617 (PMC2868894; doi:10.1371/journal.pone.0010617)
Supplement: Method S2 — Cathodic reduction potentials of colchicine, colchicides and bicolchicides. (0.02 MB DOC) [file pone.0010617.s002.doc]

**Cathodic reduction potentials of colchicine, colchicides and bicolchicides**

Cyclic voltammograms were collected using a Bioanalytical Systems CV-27 Voltammograph with a platinum disk working electrode, platinum wire counter electrode, and SCE reference electrode in dry N_2_-flushed DMF solution containing 0.1 M *n*-Bu_4_NClO_4_ as supporting electrolyte and the sample at concentration 4 ·10^-4^ M.

At scan rate 0.1 V s^-1^, colchicine gives Ep (V vs SCE) = – 1.69, while colchicide (**3**) Ep = –1.48, isocolchicide (**6**) Ep = –1.50, bicochicide (**2**) Ep = –1.28, biisocolchicide (**5**) Ep = –1.27.
