# Supplementary material for: On the Chiroptical Behavior of Conjugated Multichromophoric Compounds of a New Pseudoaromatic Class: Bicolchicides and Biisocolchicides
Source: PLoS One. 2010 May 12;5(5):e10617. doi: 10.1371/journal.pone.0010617 (PMC2868894; doi:10.1371/journal.pone.0010617)
Supplement: Calculations S1 — Quantum mechanical calculations; molecular dynamics. (0.09 MB DOC) [file pone.0010617.s003.doc]

**Quantum mechanical calculations**

**DFT/M05‑2X/6‑31G* optimized geometries (units angstrom):**

(*R*_a_,7*S*)(*R*_a_,7'*S*)-**5a**

C -0.542 -7.297 -1.495

C 0.044 -8.171 -0.579

C 0.778 -7.652 0.497

C 0.942 -6.277 0.616

C 0.348 -5.388 -0.293

C -0.400 -5.917 -1.359

C -1.048 -4.990 -2.365

C -2.253 -4.224 -1.789

C -1.962 -3.767 -0.350

C -0.591 -3.113 -0.253

C 0.513 -3.920 -0.134

O -0.030 -9.521 -0.639

O 1.304 -8.488 1.441

O 1.698 -5.797 1.653

C -0.817 -10.083 -1.675

C 2.634 -8.901 1.125

C 0.977 -5.757 2.890

N -2.997 -2.882 0.172

C -4.225 -3.379 0.472

H -2.734 -1.998 0.592

C -5.158 -2.399 1.153

O -4.559 -4.526 0.206

C -0.593 -1.685 -0.354

C 1.645 -0.897 0.518

C 2.306 -2.192 0.368

C 1.851 -3.433 0.075

C 0.267 -0.696 0.021

O 2.321 0.034 0.974

C 0.542 7.297 -1.495

C -0.044 8.171 -0.579

C -0.778 7.652 0.497

C -0.942 6.277 0.616

C -0.348 5.388 -0.293

C 0.400 5.917 -1.359

C 1.048 4.990 -2.365

C 2.253 4.224 -1.789

C 1.962 3.767 -0.350

C 0.591 3.113 -0.253

C -0.513 3.920 -0.134

O 0.030 9.521 -0.639

O -1.304 8.488 1.441

O -1.698 5.797 1.653

C 0.817 10.083 -1.675

C -2.634 8.901 1.125

C -0.977 5.757 2.890

N 2.997 2.882 0.172

C 4.225 3.379 0.472

H 2.734 1.998 0.592

C 5.158 2.399 1.153

O 4.559 4.526 0.206

C 0.593 1.685 -0.354

C -1.645 0.897 0.518

C -2.306 2.192 0.368

C -1.851 3.433 0.075

C -0.267 0.696 0.021

O -2.321 -0.034 0.974

H -1.114 -7.679 -2.328

H -0.301 -4.270 -2.705

H -1.362 -5.563 -3.238

H -3.146 -4.846 -1.757

H -2.466 -3.351 -2.411

H -1.950 -4.665 0.270

H -0.774 -11.158 -1.530

H -0.413 -9.829 -2.658

H -1.853 -9.745 -1.605

H 2.952 -9.559 1.929

H 3.295 -8.034 1.071

H 2.646 -9.443 0.178

H 1.661 -5.348 3.629

H 0.670 -6.762 3.176

H 0.105 -5.107 2.794

H -5.418 -2.794 2.135

H -4.722 -1.407 1.261

H -6.076 -2.337 0.570

H -1.531 -1.310 -0.737

H 3.369 -2.098 0.554

H 2.609 -4.204 0.042

H 1.114 7.679 -2.328

H 1.362 5.563 -3.238

H 0.301 4.270 -2.705

H 2.466 3.351 -2.411

H 3.146 4.846 -1.757

H 1.950 4.665 0.270

H 0.774 11.158 -1.530

H 1.853 9.745 -1.605

H 0.413 9.829 -2.658

H -2.952 9.559 1.929

H -2.646 9.443 0.178

H -3.295 8.034 1.071

H -1.661 5.348 3.629

H -0.105 5.107 2.794

H -0.670 6.762 3.176

H 6.076 2.337 0.570

H 4.722 1.407 1.261

H 5.418 2.794 2.135

H 1.531 1.310 -0.737

H -3.369 2.098 0.554

H -2.609 4.204 0.042

end

(*R*_a_,7*S*)(*S*_a_,7'*S*)-**5b**

C1 1.499 -7.213 -1.005

C2 0.506 -8.050 -0.493

C3 -0.706 -7.494 -0.056

C4 -0.922 -6.125 -0.186

C5 0.081 -5.274 -0.670

C6 1.294 -5.834 -1.091

C7 2.398 -4.922 -1.563

C8 3.044 -4.142 -0.402

C9 2.027 -3.621 0.626

C10 0.808 -2.995 -0.065

C11 -0.125 -3.800 -0.671

O1 0.608 -9.395 -0.378

O2 -1.661 -8.286 0.515

O3 -2.145 -5.627 0.175

C12 1.819 -9.994 -0.804

C13 -2.603 -8.784 -0.437

C14 -2.230 -5.268 1.559

N1 1.693 -4.659 1.588

C15 1.025 -4.335 2.726

H1 1.796 -5.627 1.330

C16 0.731 -5.495 3.656

O4 0.718 -3.181 2.987

C17 0.835 -1.565 -0.070

C18 -1.325 -0.768 -1.143

C19 -1.781 -2.089 -1.560

C20 -1.295 -3.337 -1.360

C21 0.005 -0.581 -0.525

O5 -2.084 0.182 -1.375

C22 -0.411 7.250 1.674

C23 0.163 8.203 0.835

C24 0.904 7.784 -0.279

C25 1.090 6.426 -0.507

C26 0.509 5.456 0.324

C27 -0.250 5.886 1.427

C28 -0.888 4.871 2.350

C29 -2.078 4.138 1.705

C30 -1.776 3.809 0.235

C31 -0.397 3.182 0.087

C32 0.696 4.008 0.044

O6 0.070 9.544 1.005

O7 1.416 8.702 -1.154

O8 1.853 6.045 -1.580

C33 -0.725 10.007 2.082

C34 2.747 9.093 -0.818

C35 1.115 6.043 -2.806

N2 -2.799 2.964 -0.367

C36 -4.024 3.476 -0.648

H2 -2.533 2.097 -0.820

C37 -4.948 2.539 -1.400

O9 -4.365 4.603 -0.314

C38 -0.383 1.748 0.059

C39 1.877 1.079 -0.875

C40 2.526 2.363 -0.591

C41 2.047 3.563 -0.192

C42 0.495 0.821 -0.410

O10 2.548 0.193 -1.402

H3 2.438 -7.622 -1.350

H4 3.164 -5.497 -2.085

H5 1.988 -4.205 -2.277

H6 3.586 -3.289 -0.814

H7 3.768 -4.765 0.130

H8 2.500 -2.832 1.208

H9 1.994 -9.814 -1.867

H10 1.702 -11.059 -0.629

H11 2.666 -9.620 -0.225

H12 -3.310 -9.394 0.120

H13 -2.097 -9.397 -1.184

H14 -3.126 -7.956 -0.918

H15 -3.252 -4.940 1.725

H16 -1.542 -4.451 1.785

H17 -2.014 -6.137 2.181

H18 0.600 -6.434 3.118

H19 1.558 -5.607 4.359

H20 -0.169 -5.264 4.221

H21 1.734 -1.176 0.390

H22 -2.700 -2.010 -2.128

H23 -1.885 -4.132 -1.797

H24 -0.991 7.554 2.534

H25 -1.214 5.366 3.266

H26 -0.132 4.136 2.633

H27 -2.279 3.212 2.251

H28 -2.981 4.746 1.722

H29 -1.773 4.757 -0.307

H30 -0.695 11.091 2.027

H31 -1.756 9.663 1.983

H32 -0.319 9.678 3.041

H33 3.053 9.820 -1.565

H34 2.767 9.552 0.172

H35 3.412 8.228 -0.847

H36 1.802 5.708 -3.578

H37 0.273 5.351 -2.735

H38 0.759 7.049 -3.029

H39 -4.493 1.571 -1.605

H40 -5.236 3.017 -2.336

H41 -5.853 2.402 -0.809

H42 -1.309 1.320 0.415

H43 3.593 2.296 -0.766

H44 2.790 4.341 -0.078

end

**Molecular dynamics**

(*R*_a_,7*S*)(*R*_a_,7'*S*)-**5a**

Parameter files in AMBER prep format for the minimized structures above

0 0 2

This is a remark line

molecule.res

UNK INT 0

CORRECT OMIT DU BEG

0.0000

1 DUMM DU M 0 -1 -2 0.000 .0 .0 .00000

2 DUMM DU M 1 0 -1 1.449 .0 .0 .00000

3 DUMM DU M 2 1 0 1.522 111.1 .0 .00000

4 O4 o M 3 2 1 1.540 111.208 180.000 -0.55802

5 C17 c M 4 3 2 1.238 56.892 26.095 0.58191

6 C18 cc M 5 4 3 1.462 117.212 -160.453 -0.23930

7 H21 ha E 6 5 4 1.083 110.443 -8.446 0.15091

8 C19 cd M 6 5 4 1.354 133.027 170.301 -0.04339

9 H22 ha E 8 6 5 1.082 115.115 -179.639 0.14955

10 C10 cd M 8 6 5 1.439 130.850 -3.334 0.00943

11 C4 ca M 10 8 6 1.486 116.945 -173.340 -0.10826

12 C3 ca M 11 10 8 1.403 120.636 48.908 0.14026

13 O2 os S 12 11 10 1.370 120.123 -0.880 -0.34446

14 C13 c3 3 13 12 11 1.432 112.686 99.746 0.11655

15 H14 h1 E 14 13 12 1.087 106.316 -179.016 0.06110

16 H15 h1 E 14 13 12 1.089 110.055 61.592 0.05614

17 H16 h1 E 14 13 12 1.092 110.042 -60.194 0.03357

18 C2 ca M 12 11 10 1.390 121.420 178.803 0.03978

19 O1 os S 18 12 11 1.366 120.050 -176.212 -0.32815

20 C12 c3 3 19 18 12 1.428 112.496 -90.255 0.11663

21 H11 h1 E 20 19 18 1.087 106.498 -179.749 0.05970

22 H12 h1 E 20 19 18 1.092 110.195 61.094 0.03019

23 H13 h1 E 20 19 18 1.091 110.239 -60.730 0.04499

24 C1 ca M 18 12 11 1.402 119.583 2.296 0.12925

25 O os S 24 18 12 1.353 115.587 178.037 -0.32044

26 C11 c3 3 25 24 18 1.417 117.280 177.024 0.11109

27 H8 h1 E 26 25 24 1.086 105.846 -178.143 0.06953

28 H9 h1 E 26 25 24 1.093 111.108 62.935 0.03139

29 H10 h1 E 26 25 24 1.092 110.931 -59.328 0.04498

30 C ca M 24 18 12 1.395 119.463 -1.731 -0.19993

31 H2 ha E 30 24 18 1.080 120.475 -179.778 0.14600

32 C5 ca M 30 24 18 1.394 120.888 0.415 -0.01587

33 C6 c3 M 32 30 24 1.514 119.857 -179.883 -0.03870

34 H3 hc E 33 32 30 1.092 108.538 -130.490 0.05469

35 H4 hc E 33 32 30 1.090 109.484 -13.885 0.05260

36 C7 c3 M 33 32 30 1.540 113.022 108.602 -0.08995

37 H5 hc E 36 33 32 1.089 111.635 -79.052 0.07674

38 H6 hc E 36 33 32 1.093 109.696 160.954 0.04611

39 C8 c3 M 36 33 32 1.538 110.479 40.275 0.14718

40 N n B 39 36 33 1.458 112.397 172.106 -0.58169

41 C14 c B 40 39 36 1.358 119.908 70.833 0.66066

42 C15 c3 3 41 40 39 1.515 114.797 173.270 -0.17174

43 H17 hc E 42 41 40 1.090 108.512 -118.754 0.06456

44 H18 hc E 42 41 40 1.089 112.788 2.938 0.06404

45 H19 hc E 42 41 40 1.089 108.378 124.718 0.07354

46 O3 o E 41 40 39 1.224 122.794 -7.224 -0.62306

47 H hn E 40 39 36 1.013 119.571 -129.196 0.33436

48 H7 h1 E 39 36 33 1.091 106.810 -70.005 0.08734

49 C9 cc M 39 36 33 1.522 110.966 47.363 -0.07308

50 C16 cc M 49 39 36 1.432 115.014 95.349 -0.06244

51 H20 ha E 50 49 39 1.080 111.868 -14.826 0.14424

52 C20 cd M 50 49 39 1.363 134.700 158.685 -0.13072

53 C41 cd M 52 50 49 1.491 116.834 -165.593 -0.12853

54 C37 cc M 53 52 50 1.363 116.834 -144.089 -0.06298

55 H41 ha E 54 53 52 1.080 113.168 7.857 0.14368

56 C30 cc M 54 53 52 1.432 134.700 -165.593 -0.07221

57 C29 c3 M 56 54 53 1.522 115.014 158.685 0.14608

58 N1 n B 57 56 54 1.458 110.836 -30.272 -0.58160

59 C35 c B 58 57 56 1.358 119.908 -164.353 0.66055

60 C36 c3 3 59 58 57 1.515 114.797 173.270 -0.17194

61 H38 hc E 60 59 58 1.089 108.378 124.718 0.07390

62 H39 hc E 60 59 58 1.089 112.788 2.938 0.06397

63 H40 hc E 60 59 58 1.090 108.512 -118.754 0.06456

64 O8 o E 59 58 57 1.224 122.794 -7.224 -0.62187

65 H1 hn E 58 57 56 1.013 119.571 -4.382 0.33319

66 H28 h1 E 57 56 54 1.091 107.906 -147.954 0.08796

67 C28 c3 M 57 56 54 1.538 110.966 95.349 -0.08979

68 H26 hc E 67 57 56 1.093 109.397 -73.495 0.04601

69 H27 hc E 67 57 56 1.089 107.309 169.276 0.07659

70 C27 c3 M 67 57 56 1.540 110.479 47.363 -0.03882

71 H24 hc E 70 67 57 1.090 109.607 162.693 0.05263

72 H25 hc E 70 67 57 1.092 108.898 -80.429 0.05475

73 C26 ca M 70 67 57 1.514 113.022 40.275 -0.01516

74 C21 ca M 73 70 67 1.394 119.857 108.602 -0.20111

75 H23 ha E 74 73 70 1.080 118.636 0.305 0.14598

76 C22 ca M 74 73 70 1.395 120.888 -179.883 0.13052

77 O5 os S 76 74 73 1.353 124.949 -179.330 -0.31935

78 C32 c3 3 77 76 74 1.417 117.280 -3.222 0.11079

79 H29 h1 E 78 77 76 1.086 105.846 -178.143 0.06987

80 H30 h1 E 78 77 76 1.092 110.931 -59.328 0.04442

81 H31 h1 E 78 77 76 1.093 111.108 62.935 0.03179

82 C23 ca M 76 74 73 1.402 119.463 0.415 0.03859

83 O6 os S 82 76 74 1.366 120.350 176.774 -0.32799

84 C33 c3 3 83 82 76 1.428 112.496 91.247 0.11660

85 H32 h1 E 84 83 82 1.087 106.498 -179.749 0.05987

86 H33 h1 E 84 83 82 1.091 110.239 -60.730 0.04451

87 H34 h1 E 84 83 82 1.092 110.195 61.094 0.03018

88 C24 ca M 82 76 74 1.390 119.583 -1.731 0.14093

89 O7 os S 88 82 76 1.370 118.457 -178.015 -0.34444

90 C34 c3 3 89 88 82 1.432 112.686 -79.947 0.11650

91 H35 h1 E 90 89 88 1.087 106.316 -179.016 0.06109

92 H36 h1 E 90 89 88 1.092 110.042 -60.194 0.03349

93 H37 h1 E 90 89 88 1.089 110.055 61.592 0.05652

94 C25 ca M 88 82 76 1.403 121.420 2.296 -0.10968

95 C31 cd M 94 88 82 1.486 120.636 178.803 0.01077

96 C40 cd M 95 94 88 1.439 116.945 48.908 -0.04316

97 H43 ha E 96 95 94 1.082 113.946 2.999 0.14938

98 C39 cc M 96 95 94 1.354 130.850 -173.340 -0.23849

99 H42 ha E 98 96 95 1.083 116.519 175.355 0.15087

100 C38 c M 98 96 95 1.462 133.027 -3.334 0.58197

101 O9 o M 100 98 96 1.238 117.212 170.301 -0.56116

LOOP

C20 C17

C9 C10

C5 C4

C38 C41

C31 C30

C25 C26

IMPROPER

C18 C20 C17 O4

C17 C19 C18 H21

C18 C10 C19 H22

C4 C9 C10 C19

C3 C5 C4 C10

C4 C2 C3 O2

C3 C1 C2 O1

C2 C C1 O

C1 C5 C H2

C6 C4 C5 C

C15 N C14 O3

C8 C16 C9 C10

C9 C20 C16 H20

C17 C16 C20 C41

C38 C37 C41 C20

C30 C41 C37 H41

C29 C37 C30 C31

C36 N1 C35 O8

C27 C21 C26 C25

C26 C22 C21 H23

C21 C23 C22 O5

C22 C24 C23 O6

C23 C25 C24 O7

C26 C24 C25 C31

C25 C30 C31 C40

C39 C31 C40 H43

C38 C40 C39 H42

C39 C41 C38 O9

DONE

STOP

Corresponding frcmod file:

remark goes here

MASS

BOND

ANGLE

n -c3-cc 66.905 110.900 Calculated with empirical approach

DIHE

cd-cd-ca-ca 1 2.550 180.000 2.000 same as X -c2-ca-X

ca-ca-cd-cc 1 2.550 180.000 2.000 same as X -c2-ca-X

IMPROPER

cc-cd-c -o 10.5 180.0 2.0 General improper torsional angle (2 general atom types)

c -cd-cc-ha 1.1 180.0 2.0 Using default value

cc-cd-cd-ha 1.1 180.0 2.0 Using default value

ca-cc-cd-cd 1.1 180.0 2.0 Using default value

ca-ca-ca-cd 1.1 180.0 2.0 Using default value

ca-ca-ca-os 1.1 180.0 2.0 Using default value

ca-ca-ca-ha 1.1 180.0 2.0 General improper torsional angle (2 general atom types)

c3-n -c -o 10.5 180.0 2.0 General improper torsional angle (2 general atom types)

c3-cc-cc-cd 1.1 180.0 2.0 Using default value

cc-cd-cc-ha 1.1 180.0 2.0 Using default value

c -cc-cd-cd 1.1 180.0 2.0 Using default value

NONBON

(*R*_a_,7*S*)(*S*_a_,7'*S*)-**5b**

0 0 2

This is a remark line

molecule.res

UNK INT 0

CORRECT OMIT DU BEG

0.0000

1 DUMM DU M 0 -1 -2 0.000 .0 .0 .00000

2 DUMM DU M 1 0 -1 1.449 .0 .0 .00000

3 DUMM DU M 2 1 0 1.522 111.1 .0 .00000

4 O5 o M 3 2 1 1.540 111.208 180.000 -0.56814

5 C18 c M 4 3 2 1.238 127.816 -13.724 0.58926

6 C19 cc M 5 4 3 1.458 116.733 33.479 -0.22026

7 H22 ha E 6 5 4 1.083 110.435 6.543 0.15391

8 C20 cd M 6 5 4 1.354 132.871 -172.507 -0.05309

9 H23 ha E 8 6 5 1.082 114.944 179.101 0.14617

10 C11 cd M 8 6 5 1.435 131.383 -1.175 0.01205

11 C5 ca M 10 8 6 1.488 115.648 -179.761 -0.11191

12 C4 ca M 11 10 8 1.402 120.166 -55.689 0.13226

13 O3 os S 12 11 10 1.369 120.631 6.061 -0.33614

14 C14 c3 3 13 12 11 1.432 113.516 -91.501 0.11600

15 H15 h1 E 14 13 12 1.086 106.213 -177.167 0.06647

16 H16 h1 E 14 13 12 1.092 110.499 64.046 0.02578

17 H17 h1 E 14 13 12 1.090 109.860 -58.388 0.05487

18 C3 ca M 12 11 10 1.392 121.220 -173.823 0.03537

19 O2 os S 18 12 11 1.366 120.056 174.886 -0.33046

20 C13 c3 3 19 18 12 1.429 112.615 88.971 0.11671

21 H12 h1 E 20 19 18 1.087 106.433 -179.937 0.06100

22 H13 h1 E 20 19 18 1.091 110.262 61.142 0.04437

23 H14 h1 E 20 19 18 1.091 110.196 -60.853 0.03345

24 C2 ca M 18 12 11 1.403 119.645 -4.002 0.12507

25 O1 os S 24 18 12 1.354 115.613 -177.415 -0.31679

26 C12 c3 3 25 24 18 1.417 117.321 -179.768 0.11024

27 H9 h1 E 26 25 24 1.092 111.097 -61.183 0.03640

28 H10 h1 E 26 25 24 1.086 105.915 179.850 0.07208

29 H11 h1 E 26 25 24 1.092 111.026 61.047 0.03777

30 C1 ca M 24 18 12 1.396 119.409 2.462 -0.20778

31 H3 ha E 30 24 18 1.081 120.544 -179.783 0.14625

32 C6 ca M 30 24 18 1.397 120.672 -0.300 -0.02144

33 C7 c3 M 32 30 24 1.508 120.595 176.146 -0.04441

34 H4 hc E 33 32 30 1.091 110.190 17.692 0.05230

35 H5 hc E 33 32 30 1.092 109.086 135.210 0.06750

36 C8 c3 M 33 32 30 1.541 112.169 -104.518 -0.08362

37 H6 hc E 36 33 32 1.091 108.630 -161.877 0.05525

38 H7 hc E 36 33 32 1.093 110.846 80.261 0.05769

39 C9 c3 M 36 33 32 1.537 113.464 -41.211 0.14051

40 N1 n B 39 36 33 1.454 110.639 83.343 -0.57422

41 C15 c B 40 39 36 1.359 119.788 166.937 0.66124

42 C16 c3 3 41 40 39 1.516 115.264 -179.950 -0.17358

43 H18 hc E 42 41 40 1.090 112.319 -30.378 0.05938

44 H19 hc E 42 41 40 1.091 109.079 90.201 0.06150

45 H20 hc E 42 41 40 1.087 108.468 -152.014 0.07843

46 O4 o E 41 40 39 1.222 121.832 -1.733 -0.61242

47 H1 hn E 40 39 36 1.007 119.549 -24.862 0.31670

48 H8 h1 E 39 36 33 1.089 108.407 -161.690 0.09760

49 C10 cc M 39 36 33 1.535 111.265 -44.451 -0.07601

50 C17 cc M 49 39 36 1.430 113.229 -102.204 -0.06027

51 H21 ha E 50 49 39 1.082 111.937 1.875 0.14099

52 C21 cd M 50 49 39 1.365 135.255 -178.786 -0.11892

53 C42 cd M 52 50 49 1.490 116.912 -175.168 -0.14840

54 C38 cc M 53 52 50 1.360 117.111 -135.116 -0.05658

55 H42 ha E 54 53 52 1.080 113.382 7.431 0.15765

56 C31 cc M 54 53 52 1.434 133.978 -166.491 -0.07691

57 C30 c3 M 56 54 53 1.522 114.998 158.223 0.14055

58 N2 n B 57 56 54 1.457 110.918 -30.171 -0.57825

59 C36 c B 58 57 56 1.357 120.034 -163.259 0.66134

60 C37 c3 3 59 58 57 1.516 114.821 173.993 -0.17251

61 H39 hc E 60 59 58 1.089 112.841 0.401 0.06066

62 H40 hc E 60 59 58 1.090 108.427 -121.444 0.06486

63 H41 hc E 60 59 58 1.090 108.360 122.103 0.07356

64 O9 o E 59 58 57 1.224 122.846 -6.511 -0.61810

65 H2 hn E 58 57 56 1.014 119.762 -1.086 0.32879

66 H29 h1 E 57 56 54 1.092 107.870 -147.627 0.10160

67 C29 c3 M 57 56 54 1.536 111.060 95.621 -0.08622

68 H27 hc E 67 57 56 1.094 109.421 -73.278 0.04765

69 H28 hc E 67 57 56 1.089 107.311 169.522 0.06185

70 C28 c3 M 67 57 56 1.539 110.545 47.566 -0.03955

71 H25 hc E 70 67 57 1.091 109.685 162.528 0.05101

72 H26 hc E 70 67 57 1.092 108.867 -80.533 0.05891

73 C27 ca M 70 67 57 1.513 112.943 40.087 -0.01800

74 C22 ca M 73 70 67 1.396 119.939 108.701 -0.20166

75 H24 ha E 74 73 70 1.081 118.530 0.349 0.14407

76 C23 ca M 74 73 70 1.393 120.957 -179.758 0.12821

77 O6 os S 76 74 73 1.355 124.965 -179.323 -0.32002

78 C33 c3 3 77 76 74 1.416 117.222 -3.172 0.11149

79 H30 h1 E 78 77 76 1.086 105.802 -178.289 0.06875

80 H31 h1 E 78 77 76 1.091 110.990 -59.421 0.04320

81 H32 h1 E 78 77 76 1.092 111.134 62.855 0.03106

82 C24 ca M 76 74 73 1.402 119.456 0.503 0.03903

83 O7 os S 82 76 74 1.368 120.372 176.542 -0.32770

84 C34 c3 3 83 82 76 1.427 112.480 92.201 0.11671

85 H33 h1 E 84 83 82 1.086 106.506 -179.711 0.05891

86 H34 h1 E 84 83 82 1.091 110.232 -60.767 0.04380

87 H35 h1 E 84 83 82 1.091 110.169 61.136 0.03016

88 C25 ca M 82 76 74 1.390 119.551 -1.982 0.14149

89 O8 os S 88 82 76 1.371 118.336 -178.018 -0.34335

90 C35 c3 3 89 88 82 1.431 112.582 -82.267 0.11642

91 H36 h1 E 90 89 88 1.086 106.447 -178.756 0.06090

92 H37 h1 E 90 89 88 1.092 110.046 -59.858 0.03519

93 H38 h1 E 90 89 88 1.090 110.021 61.824 0.05492

94 C26 ca M 88 82 76 1.403 121.533 2.519 -0.10419

95 C32 cd M 94 88 82 1.487 120.643 178.624 0.00951

96 C41 cd M 95 94 88 1.442 116.699 49.366 -0.05025

97 H44 ha E 96 95 94 1.082 113.849 3.476 0.14702

98 C40 cc M 96 95 94 1.352 130.849 -172.893 -0.23426

99 H43 ha E 98 96 95 1.083 116.835 175.771 0.14936

100 C39 c M 98 96 95 1.466 132.638 -2.829 0.57319

101 O10 o M 100 98 96 1.230 118.180 170.190 -0.53095

LOOP

C21 C18

C10 C11

C6 C5

C39 C42

C32 C31

C26 C27

IMPROPER

C19 C21 C18 O5

C18 C20 C19 H22

C19 C11 C20 H23

C5 C10 C11 C20

C4 C6 C5 C11

C5 C3 C4 O3

C4 C2 C3 O2

C3 C1 C2 O1

C2 C6 C1 H3

C7 C5 C6 C1

C16 N1 C15 O4

C9 C17 C10 C11

C10 C21 C17 H21

C18 C17 C21 C42

C39 C38 C42 C21

C31 C42 C38 H42

C30 C38 C31 C32

C37 N2 C36 O9

C28 C22 C27 C26

C27 C23 C22 H24

C22 C24 C23 O6

C23 C25 C24 O7

C24 C26 C25 O8

C27 C25 C26 C32

C26 C31 C32 C41

C40 C32 C41 H44

C39 C41 C40 H43

C40 C42 C39 O10

DONE

STOP

Corresponding frcmod file:

remark goes here

MASS

BOND

ANGLE

n -c3-cc 66.905 110.900 Calculated with empirical approach

DIHE

cd-cd-ca-ca 1 2.550 180.000 2.000 same as X -c2-ca-X

ca-ca-cd-cc 1 2.550 180.000 2.000 same as X -c2-ca-X

IMPROPER

cc-cd-c -o 10.5 180.0 2.0 General improper torsional angle (2 general atom types)

c -cd-cc-ha 1.1 180.0 2.0 Using default value

cc-cd-cd-ha 1.1 180.0 2.0 Using default value

ca-cc-cd-cd 1.1 180.0 2.0 Using default value

ca-ca-ca-cd 1.1 180.0 2.0 Using default value

ca-ca-ca-os 1.1 180.0 2.0 Using default value

ca-ca-ca-ha 1.1 180.0 2.0 General improper torsional angle (2 general atom types)

c3-n -c -o 10.5 180.0 2.0 General improper torsional angle (2 general atom types)

c3-cc-cc-cd 1.1 180.0 2.0 Using default value

cc-cd-cc-ha 1.1 180.0 2.0 Using default value

c -cc-cd-cd 1.1 180.0 2.0 Using default value

NONBON
